# No Need to Stick Together to Be Connected: Multiple Types of Enhancers’ Networking

**DOI:** 10.3390/cancers13205201

**Published:** 2021-10-16

**Authors:** Emanuele Vitale, Mila Gugnoni, Alessia Ciarrocchi

**Affiliations:** 1 Laboratory of Translational Research, Azienda USL-IRCCS di Reggio Emilia, Viale Risorgimento 80, 42123 Reggio Emilia, Italy; Emanuele.Vitale@ausl.re.it (E.V.); Mila.Gugnoni@ausl.re.it (M.G.); 2 Clinical and Experimental Medicine PhD Program, University of Modena and Reggio Emilia, Via Università 4, 41121 Modena, Italy

**Keywords:** enhancers, non-coding genome, gene expression regulation, modular organization, liquid-liquid phase separation

## Abstract

**Simple Summary:**

Transcription regulation programs require the functional interaction of distal and proximal regulatory regions, interacting by specific 3D chromatin configurations. Enhancers are cis-acting regulatory elements able to promote gene expression regardless their orientation and distance from the transcription starting site. Their systematic mapping by genome-wide chromatin profiling and chromosome conformation analysis, combined with the development of gene-editing approaches to modulate their function, revealed that many enhancers work together to fine-tune the expression of their target genes. This review aim to describe the functions of different types of enhancers and the modalities of enhancers’ interaction, focusing on their role in the regulation of complex biological processes like cancer development.

**Abstract:**

The control of gene expression at a transcriptional level requires a widespread landscape of regulatory elements. Central to these regulatory circuits are enhancers (ENHs), which are defined as cis-acting DNA elements able to increase the transcription of a target gene in a distance- and orientation-independent manner. ENHs are not independent functional elements but work in a complex and dynamic cooperative network, constituting the building blocks of multimodular domains of gene expression regulation. The information from each of these elements converges on the target promoter, contributing to improving the precision and sharpness of gene modulation. ENHs’ interplay varies in its nature and extent, ranging from an additive to redundant effect depending on contexts. Moving from super-enhancers that drive the high expression levels of identity genes, to shadow-enhancers, whose redundant functions contribute to buffering the variation in gene expression, this review aims to describe the different modalities of ENHs’ interaction and their role in the regulation of complex biological processes like cancer development.

## 1. Introduction

The human genome encodes the blueprint of human beings. Cell fates and complex body plans are established through an elaborate succession of signals that define multidimensional and precise patterns of gene expression [1]. The same happens in many diseases including cancer, in which evolution strongly relies on the aberrant reactivation of morphogenetic pathways [2]. For a long time, gene expression regulation has been considered as a mono-dimensional process in which each gene is controlled by the activity of the nearest promoter. Instead, however, systematic functional analysis of the non-coding genome has revealed that gene expression is far more complicated than expected. The precise spatiotemporal transcription of a gene needs a continuous and widespread regulatory landscape involving a specific genomic architecture and the hierarchical interactions of multiple interspersed regulatory elements [3].

Central to the precise execution of these multidimensional circuitries are the enhancers (ENHs) [4]. They have been classically described as cis-acting DNA elements that can increase the transcription of target genes by several magnitudes via their close three-dimensional space proximity with promoters [5,6,7]. Long ENHs, with hundreds of base-pairs, function regardless of their orientation and at various distances by recruiting multiple transcription factors (TFs) in a cooperative manner [1,8,9]. The binding of a single molecule of TF within the ENH triggers the recruitment of many others, favoring DNA bending and the assembly of large chromatin-remodeling complexes [10,11,12,13]. This results in chromatin structure modifications that ease the recruitment and progression of the transcriptional machinery at target promoters [14,15,16]. DNA loops bring distal regulatory elements near promoters, allowing ENHs to carry out their activating functions. ENH-promoters’ specificity is ensured by insulator elements localized within the ENH-promoters’ domain boundaries and blocks the spread of ENHs’ signals to unrelated genes [17].

Systematic mapping of ENHs by genome-wide chromatin profiling and chromosome conformation across cell types revealed that these elements outnumber genes by at least an order of magnitude, underlining the existence of multimodal cooperative, convergent and/or redundant relationships among them [3,18]. Indeed, genetic approaches have demonstrated that multiple ENHs can converge on the regulation of a single gene, and that vice versa, a single ENH may simultaneously boost the transcription of multiple targets [19,20,21]. These multiple regulatory interactions and their timing- and context-specificity dictate the execution of precise transcriptional programs defining cell-specific features.

Currently, the understanding of the complicated nature of ENHs’ interplay is limited by a lack of genome-wide strategies for the systematic study of these elements in their native genomic context. However, the available information indicates that ENHs’ interplay varies in nature and extent, ranging from complete redundancy, like shadow-ENHs during embryonic development, to tight functional interdependence, as among the elements composing super-ENHs (SEs). The type of functional interaction among ENHs seems to be dictated by the function of the target gene in the cell-specific or time-specific context. During embryogenesis, shadow-ENHs are responsible for the regulation of genes that drive morphogenesis. Their redundancy serves to ensure phenotypic robustness and to buffer potential alteration and prevent deleterious developmental consequences [22,23]. Multiple enhancers stitched together within a minimum of 12.5 Kb regions showing unusually high enrichment of transcriptional coactivators are defined as SEs. In cancer, SEs are responsible for the massive expression of specific oncogenes to which cancer cells are addicted [24,25,26].

Interaction modalities among ENHs seem to have evolved following the complexity of the system in which they operate to fulfill requirements of timely and highly differentiated expression modulations [19,22]. In cancer cells, plasticity and adaptation capacity are fundamental for survival and progression and require fast and efficient reorganization of the transcriptional landscape through multimodal and transient communication among multiple regulatory elements [27,28].

In the present work, we aim to discuss the emerging evidence about the complexity of ENHs’ interplay and their context-specific effect on transcription regulation. We also report on the main features of the ENH networks and on the models that have been proposed to explain their function.

## 2. A Journey at the Center of the Dark Genome: The Challenge of Enhancers’ Functional Characterization

In the functional study of the genome, ENHs represent a major challenge. While assigning each promoter to its target gene based on spatial proximity looks to be quite easy, far more complicated is to functionally annotate ENHs and to match them to their targets. Reporter assays, either for single or—when combined with high-throughput sequencing (MPRA, STARR-seq, and FIREWACh)—multiple ENHs, and classical mutagenesis approaches have represented for long time the most powerful tool to explore transactivation properties of ENHs and to dissect their mechanism of action [29,30,31,32]. However, when studying ENHs out of their local chromatin context, these approaches do not allow us to investigate the complexity of their functional relationships and the contribution of each of these elements within this model.

Recent technical improvements have revolutionized the ability to study ENHs, enabling us to:

(1) map ENHs and their functional status through the genome-wide analysis of specific DNA features such as the distribution of histone markers, chromatin modifiers, and TFs (ChIP-seq, DNaseI-seq, ATAC-seq) [3,33,34,35,36,37,38];
(2) detect the interactions between genomic elements in a 3D space, mapping the multi-connections established between functional elements independently of their linear distance (5C, Hi-C, ChIA-PET) [39,40,41];
(3) interfere with the function of single or multiple ENHs to evaluate the context-specific effects at a global genomic level (CRISPR-based genome editing approaches) [19,21,42,43,44].

Such information, exponentially multiplied for a massive number of different cell types and conditions, has rendered a picture of striking complexity. ENHs’ activity and their interconnections throughout the genome are dependent on highly specific modalities dictated by the cellular context and precise timing. This plasticity likely holds a large part of the secret of life and represents a source of diversification during development and complex diseases like cancer [45].

Understanding the modalities of ENHs’ cooperation on a genome-wide scale and the consequences in terms of gene expression and complex phenotypes constitutes a fundamental goal of modern biology.

## 3. SUPER-Enhancers: Get Close to Maximize the Effect

The recent characterization of SEs supports the hypothesis that transcription is founded on ENHs’ teamwork. Studying the genomic distribution of master TFs in murine embryonic stem cells (mESC), Whyte and colleagues noticed the existence of scattered clusters of consecutive ENHs [24]. At the same time, profiling the chromatin landscape and transcriptome of pancreatic islets, Parker et al. identified cell-specific long ENHs involved in the regulation of lineage-specific genes that the authors defined as stretch-ENHs [46]. Subsequent studies confirmed the existence of such elements and established their distinctive features.

SEs differ from classical ENHs in several ways. SEs have an average length that is an order of magnitude higher than canonical enhancers (10–60 Kb vs. 1–4 Kb). They are also characterized by higher levels of active chromatin markers such as H3K4me1 and H3K27ac and a higher occupancy of master TFs and RNA-PolII. In addition, SEs show an unusually high binding of chromatin organizers like BRD4 and components of the Mediator complex compared to classical ENHs [24,25,26,47]. Taking advantage of these peculiar features, Young and colleagues developed an algorithm, ROSE, that infers the localization of SEs based on the density and genomic distribution of H3K27ac, MED1, BRD4, or other factors enriched in these elements [25]. The application of ROSE, as well as other lately developed tools, to large ChIP-seq and/or ATAC-seq datasets, allowed researchers to map SEs in many cellular types and conditions in vitro and in vivo, revealing information about their functions and molecular properties [48,49].

SEs are not static genomic elements but their existence is highly context dependent [13,25,50]. SEs’ cellular specificity is related to their functions. These elements serve to control the expression of those genes that are particularly important for a specific cell type in a specific context and timing. Indeed, SEs ensure a robust level of gene expression, one that is higher than that promoted by classical ENHs [24,25,46].

Constituted by close stretches of single ENHs within a defined genomic region, SEs are organized as modular elements. Single regulatory units within SEs interact with each other and with the target transcription starting site (TSS), maximizing the transcriptional effect.

**Figure 1:**
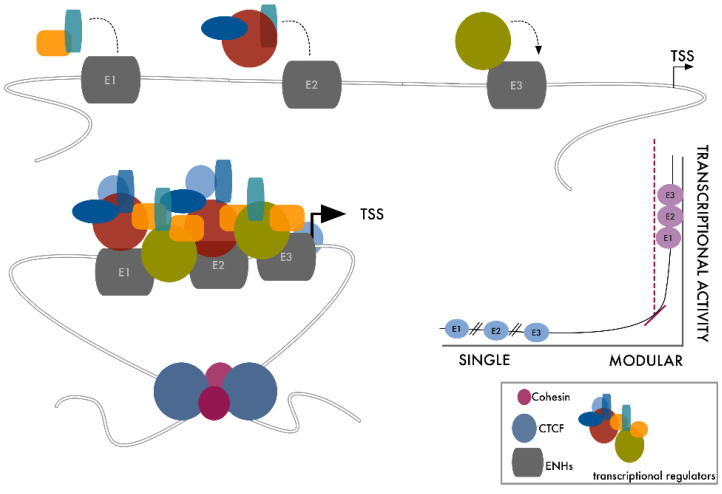
Super-ENHs are modular linear elements that boost transcription by improving the interactions among transcriptional promoting factors. Here we present a schematic representation of SE assembly. Binding of transcription factors to each of the components of the SE modifies chromatin’s accessibility, favoring the rapid recruitment of additional factors on-site, leading to the cooperative assembly of high-density protein complexes in close proximity to the target TSS. CTCF dimers and cohesin, at the boundaries of this transcriptional domain, form structural loops that isolate the system, avoiding the effect leaking to neighboring genomic sites.

Sierbsbaek et al. demonstrated that the single functional units constituting SEs have features of “TF hotspots”. Each of these elements cooperates in the constitution of SE by loading a rich repertoire of TFs (especially lineage-specific TFs), transcription activators, and co-activators (BRD4; CDK7; MED1…), concentrating a high amount of transcriptional promoting factors on a precise genomic region, which modify the chromatin structure, maximizing transcriptional efficiency [51]. In this way, SEs result in highly performant regulatory platforms able to drive gene expression at higher levels than classical interspersed ENHs [24,25] (Figure 1).

Within SEs, single regulatory modules are generally not redundant and establish complex interactions producing specific outputs [24]. Each module contains consensus sequences for specific and distinct TF subsets. SE modular organization multiplies the number of signaling possibilities, promoting and magnifying its activity. Perturbation of just one of these modules propagates on the entire complex, modifying the SE functional output. The binding of just one master TF on one of the SE modules is sufficient to recruit chromatin remodelers, triggering chromatin relaxation and facilitating the local access of additional TFs and transcriptional activators. This increases the accessibility of neighboring modules, affecting the whole region [52]. Vice versa, loss of function of just one of the regulatory modules or depletion of a single master TF is sufficient to impair SEs’ activity, as firstly demonstrated by Whyte et al., who showed how the knockout of *OCT4* affects the expression of SE-associated genes in mESC [24].

To avoid leaking to unrelated neighboring genes, SEs usually reside near their target TSS within specialized chromosome domains with features of a topologically associating domain (TAD). These SE target-domains do not establish contacts outside the TAD and appear as chromosome loops, the boundaries of which are maintained by *CTCF* dimers and cohesins [53,54,55] (Figure 1).

### 3.1. SE Plasticity

The activity of SEs appears to be of peculiar importance in highly dynamic processes like embryogenesis and cancer [25,51,52,56,57,58].

The SE landscape of a cell is a plastic entity that efficiently adapts to the current cellular needs [50]. SEs act as fast and high-throughput elaboration centers for the signals, mediating cell adaptation and/or reaction to precise stimuli [2,59]. SEs sustain the expression of driver genes, to which cancer cells become addicted and rely on for progression [50,58]. Genome-wide studies performed in several cancer settings demonstrated that genomic distribution of SE activity distinguishes cancer cells from their normal counterparts, discriminates different types of cancer, and marks functional and phenotypic differences among cancer progression stages. These studies revealed that these elements are acquired during tumorigenesis as part of the program required to sustain cancer development and progression [25,60,61,62,63,64].

The plastic use of SEs in cancer is not limited to the need for timely rewire transcription in response to specific conditions. In case of essential genes, different types of cancer use different SEs to drive their activation. The best example is the *MYC* oncogene, for which many cancer-specific SEs have been described. In T-cell acute lymphoblastic leukemia (T-ALL) *MYC* overexpression is driven by a *NOTCH1*-dependent SE located 1427 Kb downstream of the *MYC* locus [65], while in acute myeloid leukemia (AML) it is under the regulation of a *SWI/SNF*-dependent SE located 1.7 Kb downstream of the *MYC* locus [66]. Finally, in blastic plasmacytoid dendritic cell neoplasm (BPDCN) and multiple myeloma (MM), *MYC* upregulation depends on a *RUNX2*-associated and *IgH* lineage-specific SE [26,67]. In all these contexts, SE-mediated *MYC* regulation requires a *CTCF* binding site 2 Kb upstream of the *MYC* promoter to allow the interaction of the SE with the proximal cis-acting regulatory elements [68]. Interestingly, heterogeneity in super-enhancers’ repertoire is also found among the different variants of the same tumor type. Medulloblastoma is classified, based on clinical and biological features, into four subtypes, which have different SEs that mediate variant-specific patterns of gene expression [63]. Similarly, a set of *∂Np63* SEs characterizes the aggressive squamous variant of pancreatic cancer and is associated with poor survival [69].

### 3.2. Genetic Alterations and SEs

Cancer is a genetic disease that evolves through the progressive accumulation of mutations and genomic alterations. Similar to what happens for the coding part of the genome, where DNA aberrations may change the function of crucial proteins, genetic alterations may modify the functional status of a non-coding element, leading to the generation of novel SEs.

Point mutations in non-coding DNA regions can generate de-novo TF binding sites, priming their binding and the downstream cascade of events, leading to SE formation [13]. As well, new SEs can result from mutations in coding genes. Alterations in master TFs can lead to their aberrant function and to the activation of new elements, following ectopic binding on DNA. In squamous lung cancer, a somatic mutation in the *KLF5* DNA-binding domain alters the DNA-binding specificity and causes the formation of new SEs in the *FOXE1*, *NAMPT*, *EPHB3*, and *GASB* oncogenes [70].

Focal amplifications within a chromosome can result in new SEs. Amplification in the intergenic region between *KLF5* and *KLF12* was described in distinct types of tumors as responsible for the acquisition of *KLF5* SE [70].

New SEs can arise from small insertions/deletions or point mutations that create new TF binding sites. In T-cell acute lymphoblastic leukemia, an insertion in the *TAL1* locus creates a new binding site for *MYB*, with the subsequent formation of a new SE that drives *TAL1* overexpression [71].

Chromosome rearrangements, such as translocations, large deletions, or inversions, determine the relocation of the genetic loci within the genome. In many cases, these structural alterations have been shown to translocate the locus of an oncogene near to a pre-existing SE, resulting in its overexpression [13,58,72,73,74,75]. In blastic plasmacytoid dendritic cell neoplasm, the translocation t(6;8) carries the locus of the *MYC* under the control of *RUNX2* SEs [67]. The specificity of an SE to its target largely depends on the integrity of SE-domain boundaries; thus, the disruption of *CTCF* binding sites has been described as the cause of the ectopic SE-promoter interactions determining proto-oncogenes’ overexpression [55].

### 3.3. Pitfalls and Caveats of SEs 

The discovery of SEs profoundly modified our way of imagining transcription regulation and opened up new perspectives to understand the complex molecular mechanisms that govern genome function. Bringing together multiple ENHs in the same genomic space can exponentially increase the transcriptional strength of these regulatory elements through internal cooperation and boosted transcription of peculiar sets of key genes. However, after an initial phase of enthusiasm, critics questioned this model according to which the high transcriptional performance of SEs primarily relies on cooperation among single constituents, rather than on their intrinsic transcriptional capacity [47,76].

Early works demonstrated that single elements within SEs are more enriched in MED1, BRD4, and H3K27ac than classical ENHs, suggesting that the intrinsic properties of these elements could be relevant in determining SE transcriptional strength. Furthermore, luciferase assays demonstrated that fragments of SEs can drive luciferase activities more efficiently than classical ENHs, consolidating this hypothesis [26]. Finally, evidence exists that single regulatory modules within SEs are organized according to a functional hierarchy in which one or few master elements and secondary supportive units are recognized [77,78,79,80]. For example, dissecting the α-globin SE, Hay et al. demonstrated that only two of the modules that constitute this SE are required for its function, while the remaining ones have supportive or redundant activities [78]. Recent evidence from CRISPR/Cas9 genome editing approaches demonstrated that several in silico predicted SEs do not hold the expected transcriptional strength. On the contrary, classical intersperse single ENHs may display much higher transcriptional activity than expected, at a level similar to that attributed to SEs [81].

Based on this information, a question remains open: are SEs new functional paradigms in gene expression regulation, or is their activity just the result of clustering together multiple classical ENHs [47]? The truth is likely in the middle and the two hypotheses are not mutually exclusive. Still, SEs’ identification obtained by the in silico prediction models and based only on descriptive features does not provide the functional readout that is required to answer this question.

In this regard, improving our ability to functionally target these elements on a genome-wide scale will be fundamental.

## 4. From Dependency to Redundancy: No Need to Stick Together to Be Connected

**Figure 2:**
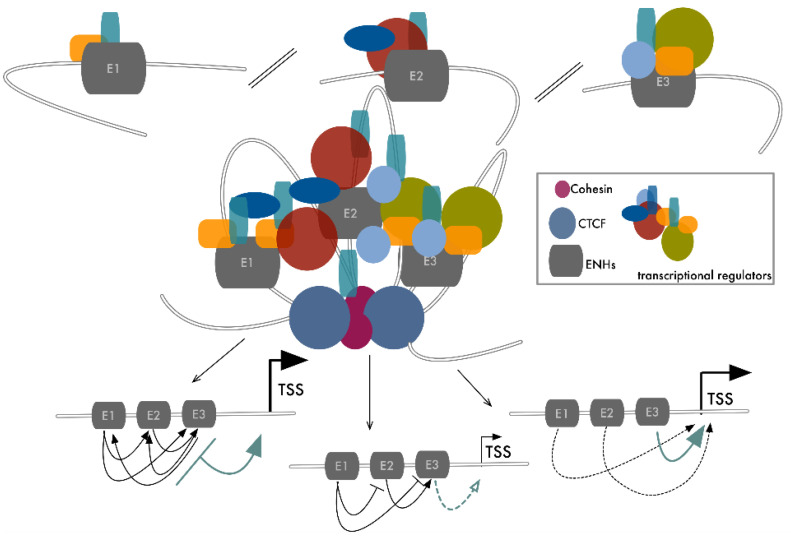
Long range-interaction among distal ENHs results in a 3D assembly similar to SE-like structures. Here we present a hypothetical model of distantly interspersed ENHs’ cooperative interaction. The loading of transcription factors to each functionally related ENH, coupled with the 3D folding of chromatin, facilitates the formation of a large and high-density complex of transcription regulators in close proximity to the target TSS. CTCF dimers and cohesin contribute to system isolation. Within this functional domain, each ENH conditions the activity of the other elements in different ways, which depend primarily on the context-specific meaning of their target gene.

**Figure 3:**
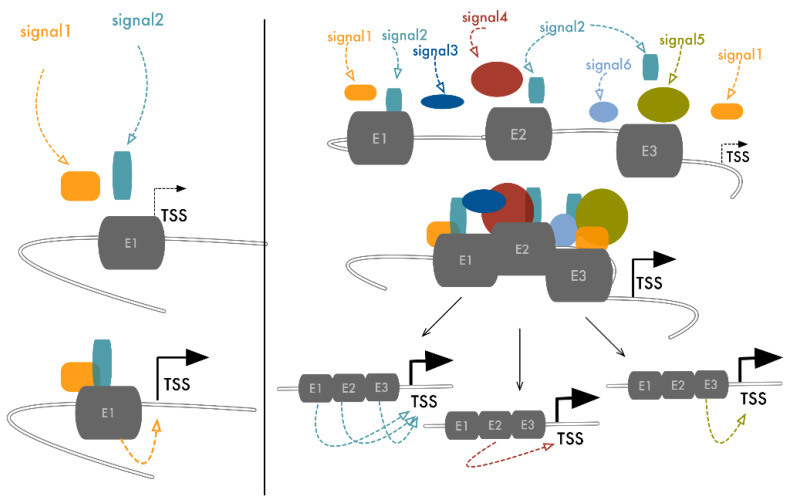
Either short-range or long-range ENHs’ interaction ensures multiple signals integrate. Binding of the TFs on a single ENH occurs upon reception of precise stimulatory signals. In a cooperative model in which multiple ENHs concur to the regulation of the same target gene, activation of just one of these elements propagates to the others, promoting the assembly of the transcriptional complex on TSS and activating transcription. In this way, the number of external signals that can activate the expression of specific target genes is amplified, improving the precision and robustness of gene modulation and the backup strategies for essential genes. This is particularly important, for example, in cancer, where cells depend on a selective panel of essential genes, the expression of which must be preserved from hostile microenvironmental perturbations.

The discovery of SEs demonstrated that closely spaced regulatory elements functionally connect to form higher-order and more efficient regulatory units. The genome is not a mono-dimensional entity but occupies a three-dimensional space in which elements, even if located at a great distance on the linear sequence, are brought together by chromatin folding [82,83,84]. Thus, in theory, this structural arrangement can organize long-range cooperative ENHs within SE-like units that maximize their functional interplay (Figure 2). As they are central for many cancers, *MYC* ENHs represent one of the best examples of plasticity and cooperation. Beside the previously described SEs, multiple additional regulatory elements have been described in a 3 Mb TAD surrounding the *MYC* gene [19,68]. Within this region, genome-wide association studies have revealed many genetic haplotypes associated with increased cancer susceptibility. Fulco and colleagues used a high-throughput CRISPRi approach to systematically map functional ENHs in this region, applying tiling sgRNAs to cover about 1.6 Mb of this domain in the K562 leukemia cell line. Seven different elements were identified, each in contact with a 3D organization *MYC* TSS and each able to transactivate it in a luciferase assay. Deletion of each one of these elements led to a strong effect on *MYC* expression and on cancer cell proliferation, indicating that each of these seven interspersed ENHs is required to sustain *MYC*’s robust expression in this setting [19]. We recently reported a similar example of reliance among different elements in the regulation of *RUNX2* expression in thyroid and breast cancer [85]. Fourteen different ENHs could be predicted around the *RUNX2* genomic locus as potential regulatory elements of this gene. A luciferase assay and histone modification profiling defined that three (ENH3, ENH11, and ENH13) of these were active and able to transactivate the *RUNX2* proximal (P2) promoter in both breast and thyroid cancer cell lines. Also, 3C experiments showed that these three ENHs interact with the *RUNX2* P2 promoter. CRISPR/Cas9-mediated depletion of these elements demonstrated that loss of function of each of them caused a dramatic drop of *RUNX2* expression without affecting syntenic SUPT3H expression. As already proven for other examples of ENHs’ cooperative interactions, the activity of these elements was coordinated by the same TF (*c-JUN*) that binds and controls each of these elements, but choosing different transcription partners. In this way, different signals can converge on the same mechanism to induce *RUNX2* upregulation [85] (Figure 3). In virtue of the established ENHs’ plasticity, we may speculate that the remaining 11 elements that were not cooperating for *RUNX2* expression in thyroid and breast cancer can be functionally relevant in other settings using different types of interplays.

ENHs’ reciprocal functional matching covers a wide range of effects, going from absolute dependency to full redundancy (Figure 2). While many examples of the combinatorial relationships among distal ENHs have been provided, the elaboration of a model that fully explains these dynamic relations is still not possible. A significant limitation to the achievement of this goal is our current inability to infer the effect of the functional alteration of a single element within the global context of the whole genome. However, the enhancement and refinement of current CRISPR/Cas9 strategies, coupled with massive sequencing, is filling this gap, highlighting how these phenomena are highly frequent and much more complex than expected. Carleton and colleagues used the estrogen signal as a model to study the combinatorial use of ENHs in gene regulation in cancer. Estrogen, through the activation of its receptor (ER), controls hundreds of genes, but the number of ER binding events along the genome is 10 times higher [86,87]. Counting the number of ER binding sites (BS) in a 100 Kb region around ER target genes, the authors showed that most positively controlled genes had more than one ER BS and that the number of these elements correlated with the strength of induction. Taking advantage of a CRISPR interference approach, based on a nuclease-deficient form of Cas9 (dCas9) fused to two repressive domains (KRAB and SID) [88,89], the authors performed simultaneous targeting of multiple ENHs converging on the same ER genes, exploring the functional effect of these inactivations. They demonstrated the existence of a complex functional hierarchy in which multiple loci bound by the same TF combine their activity to finely tune gene expression [20]. A common model emerged in which a predominant ENH is required for ER target gene expression while its activity is sustained by the remaining supportive functional elements. Supportive ENHs significantly contribute to gene expression only in the presence of the functional predominant ENH [20,21,78,90,91].

The use of loss of function approaches for the study of ENHs in their chromatin context has also revealed that multiple ENHs can cooperate additively to the simultaneous regulation of multiple genes. In K562 cells, systematic mapping of a 74 Kb region around the *GATA1* and *HDAC6* neighboring genes by CRISPRi identified two ENHs. Depletion of each of these elements determined a partial but significant drop of *GATA1* and *HDAC6* expression, demonstrating that both ENHs were required to boost the expression of both genes. Noticeably, inhibition of either the *GATA1* or *HDAC6* promoter resulted in the upregulation of the other gene, suggesting that they compete for the same ENHs and their genomic proximity can help their coordinated expression in different contexts [19]. Convergence of cooperative elements on neighboring genes is likely a mechanism to coordinate the expression of functionally related genes in vivo [92,93].

The additive models discussed above are unable to fully recapitulate the complexity of long-range ENHs’ interactions. The genome wide CRISPRi approaches revealed that some elements of the ENHs’ network have redundant functions, appearing not to be essential for the target gene’s transcriptional regulation [19,21]. In addition to the seven interdependent ENHs required for *MYC* expression in the K562 cell line, Fulco et al. identified an eighth element enriched in H3K27ac and DNase hypersensitive sites (DHS), partaking in a 3D chromosome loop with an *MYC* promoter. Targeting this element with sgRNA for CRISPRi does not impact *MYC* expression, suggesting it holds a secondary redundant role in driving *MYC* transcription [19].

Regulatory elements’ redundancy appears to be a pervasive feature of gene expression regulation, particularly evident in embryonic development [23,94]. In Drosophila, it has been estimated that as many as half of the genes driving the fate map of an adult fly are controlled by multiple ENHs with overlapping spatiotemporal activities. These elements have been historically called shadow-ENHs and they serve to improve the precision, reliability, and robustness of gene expression [95]. One of the most consolidation roles of these elements is their ability to confer phenotypic stability by maintaining the gene expression level of their targets up to a threshold level required for their functions, buffering the natural gene expression fluctuations and preventing a deleterious effect of genomic or environmental perturbations [22,96,97,98]. These elements can also suppress transcriptional noise and help foster uniform gene expression among different cell populations [99,100]. Several studies revealed that interaction among shadow- and primary-ENHs can affect gene expression in many different and context-specific ways. The synergic action of primary- and shadow-ENHs contributes to maintain a constant *Shavenbaby* expression, allowing the correct trichomes’ development in an embryo grown at suboptimal temperatures or with a reduction of *Wingless* signaling [22]. Furthermore, the presence of a semi-redundant enhancer couple preserves the spatiotemporal gene expression pattern of *SNAIL*, conferring phenotypic stability in growth temperatures of 37 °C [96]. In mammals, many developmental genes are regulated by couples of primary- and shadow-ENHs. Alterations to one of these elements can result in developmental defects and can be the cause of morphological abnormalities [23,98,101]. The deletion of a secondary *ATOH7* ENH impairs the retinal development and causes nonsyndromic congenital retinal nonattachment [102]. Little information on the use and role of shadow-ENHs in cancer is currently available. However, considering the high level of complexity of ENHs’ interplay in this context, it is likely that structural and/or functional alterations of shadow ENHs could partake in tumorigenic processes affecting the cell’s gene expression program. Indeed, shadow ENHs have been described as regulating many developmental TFs that are aberrantly reactivated in cancer. For example, shadow ENHs regulate the genes of the *HOXB* cluster [103], known for their role in several cancer types [104,105]. As well, *PAX6*, which drives stemness in lung cancer adenocarcinoma [106], has been shown to be controlled by a network of shadow-ENHs during lens development [107]. The progressive accumulation of genetic alterations during cancer progression could result in the accidental loss of function of the primary-ENHs regulating crucial genes [108]. In these conditions, shadow-ENHs could buffer potential deleterious mutations and serve to ensure the constant and strong expression of essential genes [20].

Overall, ENHs may functionally interact in many different ways that result in a variety of effects that range from additive to sub-additive (in the case of negative interference of secondary-ENHs on the primary element) to redundant. To attempt to define common rules that may explain such complexity, Bothma and colleagues developed a mathematical model proposing that ENHs’ behavior depends on the strength of their interaction with the target promoter. Weak ENHs contact a target promoter with a low frequency. Thus, they likely contribute to gene expression in an additive way to primary elements. By contrast, strong ENHs have highly frequent interactions and compete for their target promoter, thus resulting in a sub-additive relationship [109]. The same couple of regulatory elements can produce different outputs in terms of gene expression on their common target based on the cellular context. This property is well-exemplified by the Drosophila Melanogaster *Kruppel* pair of ENHs, the interaction modalities of which switch along the anterior-posterior axis of the embryo. While these two regulatory elements act anteriorly in a synergic manner, posteriorly, the distal shadow ENH attenuates the primary one, drawing the posterior boundary of the *Kruppel* gene expression profile [110,111].

## 5. Transcriptional Control Is a Matter of Density

Considering the organizational differences, proximal or long-range ENHs’ interactions rely on the same foundation and achieve the same functional output (Figure 3). Either way of communication aims to increase the number of occasions of physical contact among separate elements, improve the strength and precision of regulation, and provide backup strategies, thus ensuring transcriptional bursting on crucial genes.

ENHs are organized as modular elements hosting many sequential binding sites and loading many molecules of cooperative transcription regulators that are themselves organized as multi-domain modular structures [1,112,113].

**Figure 4:**
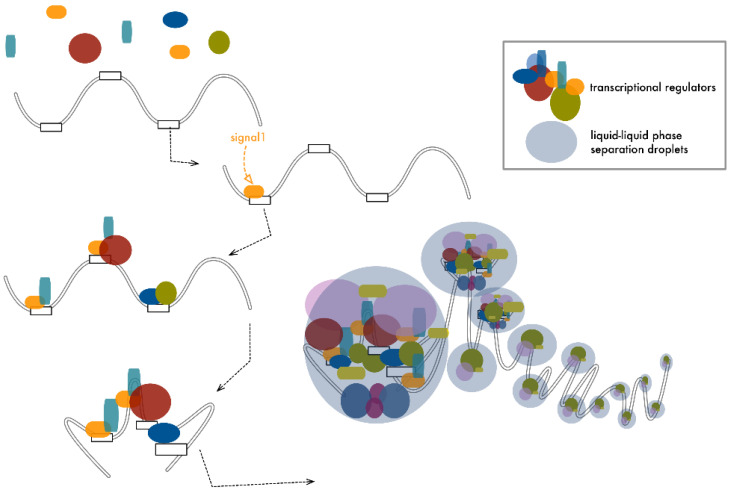
ENHs’ cooperation increases the density of local contacts, promoting the formation of droplet-like membraneless organelles. In a cooperative model, long-range or short-range interactions among multiple ENHs create a multimodular organization in which a number of distinct modules and their loaded factors are brought in close proximity. Within these structures, the number of reversible contacts among proteins and between proteins and DNA increases significantly. This high-density concentration of disordered interactions promotes a liquid-phase transition, giving rise to droplet-like domains within which functional interactions are maximized, boosting transcription initiation. Similar structures have also been proposed for other transcription-associated processes like elongation and splicing, defining DNA as a long stretch of functional pearls.

Bringing together several ENHs drastically amplifies the spatial density of these functional modules, increasing the number of occasions od protein-protein or protein-nucleic acids’ cooperative contact [24,25,26]. Recent findings revealed that this massive concentration of intramolecular interactions and their intrinsic disordered nature engender drastic changes in the physical condition of the local environment, inducing a liquid-liquid phase-separation and giving rise, at the site of transcription, to droplet-like membraneless organelles [114,115,116,117] (Figure 4). Phase separation would achieve two distinct functional outputs. First, it would create an isolated microdomain that separates the system from the outside, contributing to isolate the effect of ENHs on specific target promoters without perturbing their environment. Second, it would maximize the interactions within the system, increasing the number and quality of the contacts with the target promoter. This could facilitate the access to the TSS and improve the loading of the transcriptional machinery on the TSS, increasing transcriptional efficiency [115,118].

Hnisz and colleagues proposed a mathematical model to explain SE assembly and function based on the number and valency of the interactions among components. According to this model, phase separation is a function of valency that expresses the number of residues in each molecule taking part in the SE assembly, which can potentially be modified and/or engaged in a cross-link with other components [119]. The performance of SEs increases at there are more dynamic interactions within components, explaining how nucleation of the SE is achieved and providing a rationale to explain how the modular organization of these elements is central to mediating their uncommon transcriptional activity [117,120]. A similar model may easily apply to long-distance ENHs’ cooperation, where the number of crosslinks among different components is promoted by chromatin looping that brings interspersed but cooperative units in close 3D proximity [54].

This liquid-liquid phase separation model could also provide an explanation for the way the same ENH can promote the synchronous boosting of two separated promoters, conceptualizing the incorporation of both target promoters within the same droplet-assembly triggered by the regulatory unit.

Cramer extended this concept, theorizing that not just transcription initiation but also elongation occurs through transient condensates. The machinery that allows progression of RNA-PolII along the gene body during transcription is a high-density complex in which the reciprocal interaction between proteins and nucleic acids promotes a phase separation similar to the one that occurs at the TSS [115]. RNA-PolII can switch between initiation and elongation condensates in a phosphorylation-dependent fashion, rationalizing transcriptional organization and regulation. In this way, the genome can be imagined as a pearl necklace where each pearl constitutes a separated functional microsystem (Figure 4).

## 6. Conclusions

The genome is an operating platform. It receives multiple signals from the surrounding environment and integrates them to generate adequate responses in a very short time with highly efficient modalities. Within this system, cooperation between ENHs is the operating language that allows the precise execution of the program, guaranteeing plasticity of the system and rigor in the implementation of procedures. As the Ancient Romans used to say, “*Vis unita fortior*”, that is “union is strenght”.
